# Oligometastatic RCC: Challenges and Emerging Therapeutic Strategies

**DOI:** 10.3390/cancers18121956

**Published:** 2026-06-16

**Authors:** Calliope Stavrou, Monica Thet, Kieran Sandhu, Shankar Siva, Cristian Udovicich, Nathan Lawrentschuk, Marlon Perera

**Affiliations:** 1 Department of Urology, Royal Melbourne Hospital, Melbourne, VIC 3050, Australia; 2 Peter MacCallum Cancer Centre, Melbourne, VIC 3000, Australiamarlon.perera@petermac.org (M.P.); 3 University of Cambridge, Cambridge CB2 1TN, UK; 4 Sir Peter MacCallum Department of Oncology, University of Melbourne, Parkville, VIC 3010, Australia; 5 Department of Radiation Oncology, GenesisCare, Melbourne, VIC 3065, Australia; 6 Department of Surgery, University of Melbourne, Parkville, VIC 3010, Australia; 7 Department of Urology, Austin Hospital, Heidelberg, VIC 3084, Australia

**Keywords:** oligometastatic disease, renal-cell carcinoma, stereotactic ablative body radiotherapy, metastasis-directed therapy

## Abstract

**Background/objectives**: Renal-cell carcinoma (RCC) accounts for approximately 4% of all solid tumours worldwide. Oligometastatic RCC, frequently defined as the presence of fewer than five metastatic lesions, is increasingly recognised as a clinically and prognostically distinct disease state, yet standardised treatment algorithms remain lacking. **Method**: This narrative review summarises current local and systemic treatment strategies for oligometastatic RCC and identifies patient populations most likely to benefit based on 26 studies published within the past ten years. **Results**: Stereotactic ablative radiotherapy (SABR) was the most frequently evaluated local modality, consistently demonstrating high local control rates with favourable toxicity profiles. Systemic therapies showed mixed efficacy and greater treatment-related adverse events, while evidence for radiofrequency ablation, cryoablation, and metastasectomy remains limited but suggests feasibility in selected patients. **Conclusions**: Overall, current evidence supports local therapy—particularly SABR—in appropriately selected patients with oligometastatic RCC, though most available evidence is retrospective and concentrated in favourable-risk ccRCC populations, limiting generalizability. Further prospective research is needed to refine patient selection criteria and optimise treatment sequencing.

## Simple Summary

This article summarises recent research on treatments that target kidney cancer that has spread to a small number of sites in the body (fewer than five). Most available evidence supports the use of targeted radiotherapy, which appears to be both safe and effective in carefully selected patients. Whole-body (systemic) treatments, such as drug therapies, show mixed results and are often associated with more side effects. However, when combined with radiotherapy, these treatments may enhance the body’s immune response against cancer at sites outside the treated area. Newer local treatment options, including cryotherapy and radiofrequency ablation, use extreme cold or heat to destroy cancer cells and may be suitable alternatives in selected cases. Overall, localised treatments are emerging as important options for managing limited metastatic kidney cancer, though further research is needed to define the best treatment combinations and patient population.

## 1. Introduction

Renal-cell carcinoma (RCC) accounts for 2% of all cancers diagnosed worldwide [1,2,3,4,5]. Global incidence has been increasing, but overall mortality is declining, attributed to increased rates of incidental diagnosis in abdominal imaging facilitating earlier treatment, alongside recent advances in systemic therapy [6]. Major histopathological subtypes include clear-cell RCC (ccRCC) (75–80%), associated with worse prognosis, as well as papillary (10–15%), and chromophobe (5%) RCC; the remaining subtypes are rare, with less than a 1% total incidence [2,7]. Approximately 10% of patients are found to have metastatic disease at the time of diagnosis, and an additional 10% of those initially treated for localised RCC with curative-intent nephrectomy subsequently develop metastatic disease, most commonly in the lungs (70%), lymph nodes (45%) and bones (32%) [8].

Between localised and polymetastatic disease exists a distinct state known as oligometastatic disease [9]; this reflects the presence of a limited number of metastatic deposits, with a distinct prognostic profile compared with polymetastatic states [9]. The ESTRO–ASTRO consensus further defines oligometastatic disease as the presence of one to five metastatic lesions, all of which are amenable to safe local treatment [10], and it may be further classified based on the timing of metastatic presentation and the status of the primary tumour, including synchronous and metachronous oligometastases, oligorecurrence during a systemic therapy-free interval, and oligoprogression occurring in the setting of otherwise controlled polymetastatic disease while on systemic therapy [10].

Patients with oligometastatic disease may have comparable survival to those with non-metastatic disease if treated with aggressive therapy [11]. Although the biological basis of the oligometastatic phenotype remains incompletely understood, emerging molecular data support its distinction from widely disseminated metastatic disease and suggest that oligometastatic RCC may represent a biologically less aggressive state, thus providing a rationale for metastasis-directed treatment strategies.

The reported approaches to treatment for oligometastatic RCC range from localised therapies, including stereotactic ablative body radiotherapy (SABR), radiofrequency ablation (RFA) and cryoablation, to systemic therapies such as programmed cell death protein 1 (PD-1) inhibitors, cytotoxic T lymphocyte-associated protein 4 (CTLA-4) inhibitors and tyrosine kinase inhibitors (TKIs). Despite increasing recognition of this disease state, there remains no standardised treatment regimen, and optimal patient selection and sequencing of therapies represents an area of active investigation [5].

The oligometastatic state also remains biologically incompletely characterised, and potential prognostic or predictive biomarkers to guide patient selection for local therapy remain in early development, lacking prospective validation in clinical trials. This biological uncertainty has important implications for patient selection in oligometastatic RCC, where the absence of validated molecular classifiers means that treatment decisions continue to rely on factors such as lesion number, site and disease-free interval rather than on intrinsic tumour biology.

This narrative review aims to examine the current evidence for various treatment strategies in the management of oligometastatic RCC, with a particular focus on metastasis-directed therapies based on 26 included studies, summarised in Table 1 below.

## 2. Methods

A structured literature search was performed using the PubMed/MEDLINE database.

The search strategy combined terms relating to oligometastatic disease, RCC, and therapeutic management, using Boolean operators as follows:

(Oligometastasis OR oligometastatic) AND (Renal cell carcinoma OR kidney neoplasm*) AND (Therap* OR management* OR treatment*). Search terms were applied to titles and abstracts. Filters were used to limit results to human studies, English-language publications, and articles published within the last 10 years to capture evidence relevant to contemporary clinical practice. Case reports and case series were excluded using publication type and title-based filters.

This search yielded 124 articles, which were screened for relevance by the two primary authors. Studies were eligible if they reported treatment strategies for oligometastatic RCC, including locally directed therapies and/or systemic treatments. Studies were excluded if they did not explicitly define oligometastatic, oligorecurrent, or oligoprogressive disease, failed to report outcomes specific to these disease states, or focused on widespread metastatic disease, locally advanced non-metastatic disease, or mixed cancer populations without RCC-specific analyses.

## 3. SABR

SABR was the most frequently evaluated treatment modality. Substantial heterogeneity was observed in radiation dose and fractionation, with no standardised protocol identified. Reported regimens ranged from single-fraction treatments of 5–25 Gy to multi-fraction schedules comprising 1–10 fractions, with total doses spanning 16–75 Gy [17,18,23,24]. Hypofractionated regimens achieving biologically effective doses (BEDs) greater than 90 Gy were commonly reported, although dosing varied both between and within studies [30].

The impact of dose and fractionation on oncological outcomes remains uncertain and conflicting across retrospective studies. In one study of 48 patients treated with SABR for extracranial metastatic lesions (48 patients and 57 lesions), the dose per fraction was not significantly associated with lesion PFS (*p* = 0.052); instead, lesion PFS was strongly predicted by early radiological response following treatment, with early tumour size reduction predicting improved outcomes (*p* < 0.0001) [13]. Conversely, a larger retrospective cohort of 87 patients encompassing 284 treated lesions demonstrated superior local control among lesions receiving a BED exceeding 90 Gy compared with those receiving ≤90 Gy (83% vs. 65%; *p* = 0.03) [34], suggesting a dose–response relationship for local control, although without a corresponding effect on OS.

Fractionation schedule has also been implicated in outcomes. In patients with bone-only oligometastatic disease, single-fraction SABR was associated with prolonged PFS (27.4 vs. 12.6 months; *p* = 0.001) and improved OS (57.2 vs. 34.4 months; *p* = 0.02) [30], although approximately half of patients received concurrent systemic therapy, which may have influenced outcomes. In contrast, another retrospective study found that repeated courses of SABR, rather than dose or fractionation, were associated with superior OS (median: 90.5 vs. 21.7 months; *p* = 0.034) [20]. This cohort included both intracranial and extracranial disease across multiple organ sites, variable dose and fractionation regimens, and universal systemic therapy. Collectively, these conflicting findings likely reflect differences in patient selection and metastatic site, and further prospective studies are required to identify an optimal dosing and fractionation schedule for specific patient populations and disease locations.

Reported oncological outcomes following SABR varied widely across studies. The median PFS ranged from 7.6 to 28.9 months [20,28], with 1-year PFS rates between 41.8% and 82.6% [23,26]. Local control rates ranged from 76% to 100%, with higher rates generally reported in smaller cohorts [23,24,34]. Despite excellent local control rates, distant disease progression was observed in almost half the patients with bone metastases within 13 months of receiving SABR [30]. The median OS ranged from 28.4 to 49.2 months [13,17], with reported 1-year OS rates between 78% and 100% [17,27]. Median follow-up durations ranged from 10.4 months to over five years [22,23,32,34].

Treatment-related toxicity was commonly assessed using Common Terminology Criteria for Adverse Events (CTCAE). Grade 1–2 adverse events (AEs) were most frequently reported, occurring in 6–63% of patients [13,22,27,32,33,36]. Grade ≥ 3 toxicity was uncommon; most studies reported no grade 3–4 AEs, though one small cohort (*n* = 30) reported grade 3–4 toxicity in 13% of patients [33], and another reported a single grade 5 AE (death), with all remaining events limited to grade 1–2 severity [23].

One retrospective study directly compared SABR with conventional fractionated radiotherapy in patients with oligometastatic RCC [36]. The study identified 60 metastatic lesions across 34 patients using PSMA PET/CT; of the 56 lesions treated with radiotherapy, 38 received SABR and 18 received conventional fractionated radiotherapy, while four lesions were managed surgically. Across all patients, freedom from local progression (FFLP) at 1, 3, and 5 years was 94%, 85%, and 85%, respectively. SABR was associated with superior local control, with local progression observed in 5% versus 28% of treated lesions, respectively (*p* = 0.043). PFS declined over time, with 1-, 3-, and 5-year rates of 47%, 26%, and 8%, while the corresponding OS rates were 88%, 71%, and 64%. The small number of surgically treated lesions precluded formal comparative analysis between radiotherapy and surgical approaches.

Beyond treatment-related factors, metastatic burden and disease distribution substantially influence outcomes following SABR. While lesion PFS does not appear to be significantly influenced by metastatic site [13], OS varies considerably by location. Lung and lymph node metastases have been associated with more favourable survival outcomes [19], whereas intracranial metastases are consistently linked to poorer prognosis [19,20]. Bone metastases have been associated with significantly shorter freedom from systemic therapy (FST) compared with non-bone metastases (8.8 vs. 40.1 months; HR: 2.21; log-rank *p* = 0.03) [37]. Among bone metastases, however, site-specific differences are notable: spinal metastases demonstrated markedly improved outcomes compared with non-spinal bone lesions (median: OS 67.8 vs. 20.3 months; *p* < 0.001) [30]. Lesion size further influenced outcomes, with metastases smaller than 14 mm associated with improved lesion PFS [13]. Consistent with these findings, increasing metastatic burden was associated with worse OS across multiple studies [19,20,26], with one study reporting a median PFS of 14.0 months in patients with a single metastasis compared with 4.1 months in those with two to four metastases [20]. These findings collectively reinforce the importance of careful patient selection when considering aggressive local therapy.

Most included studies did not stratify outcomes based on systemic therapy exposure, including prior, concurrent, or systemic therapy-naïve status [32,33], representing a significant potential confounder in the contemporary treatment landscape. Among studies that did perform subgroup analyses, findings were inconsistent. One retrospective multi-institutional study found no significant difference in lesion PFS between patients who continued systemic therapy (including TKIs or other agents) during SABR and those who temporarily interrupted treatment [13]. Conversely, another multi-institutional retrospective study reported 100% local control and OS at 12 and 18 months among patients receiving immune checkpoint inhibitors concurrently with SABR [18]. While these results raise the possibility of a synergistic interaction between SABR and immunotherapy—potentially mediated through immunomodulatory mechanisms including the abscopal effect—differences in systemic agents, patient selection, and limited cohort sizes preclude definitive conclusions, underscoring the need for prospective evaluation.

## 4. Surgical Metastasectomy

Surgical resection forms the cornerstone of curative-intent treatment for RCC, with cytoreductive nephrectomy conferring a significant improvement in OS and cancer-specific survival over non-surgical management in metastatic RCC [11]. Historically, CN was established as standard of care based on two randomised trials—the SWOG 8949 and EORTC 30947 trials—which demonstrated a survival advantage with nephrectomy followed by IFN-alfa. However, the recent advent of effective systemic agents has fundamentally challenged this, with the CARMENA trial, a phase III randomised study, demonstrating that sunitinib alone was non-inferior to CN followed by sunitinib in patients with synchronous metastatic ccRCC, leading to the conclusion that upfront CN should not be considered standard of care in patients requiring systemic therapy. Importantly, subgroup analyses identified a nuance often overlooked in broad generalisations: CN may still confer benefit in patients with only one IMDC risk factor, while the number of metastatic sites alone was not found to be a reliable determinant of surgical candidacy. Notably, patients in the sunitinib-alone arm who subsequently underwent secondary nephrectomy achieved a median OS of 48.5 months, compared with 15.7 months in those who never underwent CN, lending support to a deferred surgical strategy in selected responding patients.

The SURTIME trial addressed the surgical timing; although 28-week progression-free rates were similar between immediate and deferred CN arms, the intention-to-treat OS hazard ratio favoured deferred CN (HR: 0.57; 95% CI: 0.34–0.95; *p* = 0.03), with a median OS of 32.4 months in the deferred arm versus 15.0 months in the immediate CN arm. A post hoc analysis attributed part of this benefit to impaired delivery of systemic therapy in the upfront surgery group. Immediate CN was associated with reduced sunitinib exposure, delayed treatment onset and less profound metastatic disease control compared with the deferred approach, suggesting that early progression to surgery before systemic therapy had taken effect disadvantaged a proportion of patients. Taken together, CARMENA and SURTIME shift the evidence base towards a deferred or selective approach to CN, in which systemic therapy is initiated first to identify patients with chemosensitive disease and adequate performance reserve before surgery is considered.

An increasingly older and comorbid patient population has necessitated consideration of less invasive alternatives. In this review, surgical management was included as a comparator or treatment component in only two studies [38,39].

In a retrospective comparison of SABR (*n* = 57) and surgical metastasectomy (SM; *n* = 30), no significant difference in median OS was observed between the two modalities (40 months for SABR vs. 53 months for SM) [34]. Local control of extracranial metastases was also comparable, at 76% following SABR and 67% following SM (*p* = 0.15). For intracranial disease, initial local control following SABR was high (96%); however, approximately 46% of patients experienced intracranial relapse after a median of 5 months. In contrast, all patients treated with surgical metastasectomy for intracranial metastases relapsed, with a median time to recurrence of 7 months (range: 2–25 months) [34]. These findings suggest that while both approaches offer high initial local control, SABR may provide more durable intracranial disease control than surgery, though interpretation is limited by small sample sizes and retrospective design.

In contrast, a retrospective review of 42 patients receiving heterogeneous multimodal treatments for oligometastatic RCC with bone metastases found that complete metastasectomy was associated with significantly improved long-term survival, with a 5-year OS rate of 20.1% compared with 0% in those without complete metastatic clearance (*p* = 0.042) [16], and a retrospective study of 138 patients undergoing metastasectomy at a single organ site found that removal of metastases was associated with significantly improved survival compared with incomplete or no surgery [40].

No significant improvement in long-term OS was observed with systemic therapy, radiotherapy, or antiresorptive agents in this cohort, suggesting that in selected patients with bone-predominant disease, aggressive surgical clearance may confer a meaningful survival advantage, though the retrospective and heterogeneous nature of available data limits generalisation.

## 5. Systemic Therapy With or Without Local Treatment

Systemic therapy remains a cornerstone in the management of metastatic RCC, with PD-1 inhibitors demonstrating improvements in PFS and OS in advanced ccRCC, both as monotherapy and in combination with CTLA-4 inhibitors or tyrosine kinase inhibitors (TKIs); however, their role in oligometastatic disease remains less clearly defined. Several studies evaluated systemic therapy either alone or in combination with metastasis-directed local treatments, with outcomes varying substantially across studies due to heterogeneity in patient selection, disease burden, and treatment sequencing.

A large phase III randomised trial by Allaf et al. (2024) evaluating peri-operative nivolumab in 819 patients with high-risk or node-positive RCC did not demonstrate an improvement in recurrence-free survival, including in post hoc analyses of patients who received most of the intended treatment [12]. Treatment was associated with substantial toxicity, with 22% discontinuation due to adverse effects and higher rates of grade 4–5 toxicity compared with surgery alone. Although an oligometastatic subgroup was defined, no specific outcomes were reported for this group, limiting applicability.

Earlier studies suggest that selected patients with limited metastatic burden may derive durable benefit from high-dose interleukin-2 (IL-2) therapy. A retrospective cohort study of 145 patients with oligometastatic ccRCC treated with high-dose IL-2 reported an overall response rate of 42.8%, exceeding rates typically reported in unselected metastatic RCC populations (14–20%) [14]. The median OS was 49.4 months, increasing to 58.1 months in a predefined favourable histopathological subgroup. The most favourable outcomes were with IL-2 in patients with three or fewer metastases, with 76% remaining relapse-free at a median follow-up of 39 months, and some patients achieving long-term disease-free survival following resection of oligometastatic recurrence [14].

These findings highlight the potential for durable responses in carefully selected oligometastatic patients, despite IL-2 having largely fallen out of favour given the more predictable efficacy of VEGF-targeted therapies.

Emerging evidence suggests that combining systemic therapy with local ablative approaches may enhance outcomes. In a retrospective cohort of 100 patients with spinal metastases undergoing stereotactic radiosurgery (SRS), concurrent first-line TKI therapy was associated with markedly lower rates of local failure compared with radiosurgery alone or delayed systemic therapy, with a 12-month local failure rate of 4% versus 57% in patients treated with TKI alone [29]. Similarly, patients with bone oligometastases treated with sunitinib demonstrated better survival (30.1 months) compared with those with multiple metastases (12.7 months) [25].

A small prospective phase I/II study involving 30 patients with oligometastatic ccRCC treated with SABR to all metastatic sites followed by pembrolizumab showed promising results: the overall response rate was 63%, the 1-year PFS was 60%, and the 1-year OS was 90%, with an acceptable safety profile [33]. Although safety was the primary endpoint, this multimodal approach demonstrated feasibility and efficacy, warranting further investigation in randomised trials. One recent large-scale phase 3 trial investigating adjuvant pembrolizumab after surgical resection of oligometastatic RCC showed promising overall survival in the treatment arm (89.7%) compared with the placebo (78.0%), with a statistically significant disease-free survival for adjuvant pembrolizumab; however, the oligometastatic disease subgroup sample size was insufficient for hypothesis testing. Pembrolizumab was also associated with much higher rates of grade 3–4 AEs compared with the placebo (18.6% vs. 1.2%) in this trial [41].

Local therapy was also used to defer systemic treatment in a subset of patients with synchronous metastatic RCC and incompletely resectable disease [15]. In this study, 10 of 40 patients (25%) received additional local treatment at the time of disease progression—targeting the most rapidly progressing lesion while other metastases remained stable—using modalities including external beam radiotherapy, radiofrequency ablation, and metastasectomy. Although the incremental delay attributable specifically to local therapy was not quantified, the overall median time to targeted systemic therapy was 16 months, substantially exceeding the median time to progression of 6 months [15].

Overall, while systemic therapies offer clear benefit in advanced RCC, their optimal integration in the oligometastatic setting remains uncertain. Current evidence suggests benefits may be maximised in combination with local therapies, though prospective comparative data are lacking.

## 6. Thermal Ablative Techniques

Thermal ablative techniques, including radiofrequency ablation (RFA) and cryoablation, represent minimally invasive options for selected patients with small-volume metastatic RCC, typically lesions smaller than 3 cm [8]. These approaches are associated with low peri-procedural morbidity and favourable local control rates, but are limited by tumour size, location, and technical factors such as heat-sink effects [42]. Compared with surgical metastasectomy, ablative therapies offer reduced procedural risk, particularly relevant to the increasingly frail cancer population, though potentially at the expense of higher local recurrence, emphasising the importance of careful patient selection and oncological surveillance.

Evidence supporting RFA in metastatic RCC remains limited to a single retrospective study evaluating 53 patients who underwent RFA for pulmonary metastases, all of whom had previously undergone nephrectomy [22]. Reported OS rates at 1, 3, and 5 years were 94%, 74.5%, and 62%, respectively, comparable to surgical lung metastasectomy. Local control was high at 91%, and treatment-related toxicity was low, with grade 3–4 complications occurring in only 3% of patients, most commonly pneumothorax. However, interpretation is limited by heterogeneous systemic therapy exposure, prior thoracotomy in a subset of patients, and the presence of controlled extra-pulmonary metastases, all of which may confound survival outcomes.

Evidence for cryoablation is similarly restricted to a single retrospective series of 40 patients treated for 50 RCC bone metastases, all deemed unsuitable for surgical resection [21]. Only 25 patients were classified as oligometastatic, with the remainder having more diffuse disease; cryoablation was indicated for bone metastases growing disproportionately relative to otherwise well-controlled systemic disease. The overall local tumour control rate was 82%, increasing to 96% among patients with oligometastatic disease, comparable to outcomes reported for SABR and surgical resection. Treatment-related morbidity was low, with no procedure-related deaths and only four grade 3–4 adverse events, all of which resolved with medical management. Cryoablation may be particularly suited to bone metastases owing to improved margin visualisation and penetration through cortical bone [21]. Notably, 70% of patients had received prior systemic therapy, and treatment was delivered without interruption to concurrent systemic therapy in 15 of 21 patients receiving both simultaneously, supporting the feasibility of integration with ongoing systemic treatment.

Nevertheless, the interpretation of both RFA and cryoablation outcomes is limited by small sample sizes, heterogeneous disease burden, and extensive concurrent systemic therapy use, which precludes clear attribution of oncological benefit to ablation alone. While the available data suggest that thermal ablation can be safely integrated with systemic therapies in non-surgical candidates, robust comparative or prospective evidence in oligometastatic RCC is lacking.

## 7. The Role of Imaging

Accurate identification of true oligometastatic disease is critical to the success of metastasis-directed therapy (MDT), yet conventional imaging may underestimate metastatic burden. PSMA PET/CT has emerged as a highly sensitive imaging modality for detecting metastatic lesions because PSMA is highly expressed in both prostate cancer and RCC, particularly within the neovasculature of clear-cell tumours [43]. In the oligometastatic setting, PSMA PET/CT has demonstrated improved sensitivity over conventional imaging, detecting more than 80% of metastatic sites and identifying additional metastases in approximately 25% of patients [44]. Udovicich et al. (2025) found that PSMA PET/CT identified 12% of metastases that were not visible or equivocal on conventional CT, including lesions in bone, lung, and lymph nodes [36]. This improved detection rate enabled more accurate staging and informed the delivery of MDT to all PSMA-avid sites, with potential to optimise patient selection and, thus, permit comprehensive treatment of mRCC.

## 8. Guidelines

Current international guidelines recognise oligometastatic RCC as a unique clinical state, supporting the use of local metastasis-directed therapy, with definitions and recommendations summarised in Table 2. The EAU recommends metastasectomy or SABR for bone or brain metastases and suggests observation for unresectable oligometastases prior to systemic therapy [45]. Similarly, the National Comprehensive Cancer Network (NCCN) and Kidney Cancer Research Network of Canada guidelines endorse metastasis-directed therapy—via metastasectomy, radiotherapy, or ablative techniques, including SABR—in oligometastatic or oligoprogressive disease [46,47]. The AUA also supports consideration of surgical or ablative approaches following appropriate disease staging in selected patients; however, there is no direct mention of preferred management of oligometastatic disease [43].

The ASCO recommends local therapy in carefully selected patients: those with favourable/intermediate risk, good performance status, and limited, metachronous disease without high-risk metastatic sites. Complete metastasis-directed therapy (surgery or stereotactic radiotherapy) can be offered to achieve disease control and delay systemic therapy, with adjuvant immunotherapy considered in selected patients with clear-cell histology. For patients unsuitable for upfront local therapy, standard systemic therapy is recommended, while in oligoprogressive disease, local ablative treatment to progressing lesions may be used to prolong the benefit of ongoing systemic therapy [44].

Guideline recommendations in oligometastatic RCC are predominantly consensus-based rather than derived from prospective randomised evidence, reflecting the paucity of phase III trial data in this specific population. No randomised controlled trials have established the superiority of metastasectomy, and prospective data specifically in the oligometastatic setting remain scarce across all local treatment modalities. As such, guideline statements represent expert opinion informed by retrospective and single-arm data, and “best practice” in this context is shaped by institutional expertise, multidisciplinary team composition and local resource availability and should, therefore, be considered in the context of the individual patient circumstances.

## 9. Future Directions

There are a small number of active and recruiting trials currently investigating oligometastatic RCC; the majority are considering various combinations of localised and systemic therapy, such as the ASTROs trial (NCT06004336), which aims to determine whether 1 year of additional pembrolizumab can improve RCC control after definitive radiotherapy, or STROKER (NCT06726421), which aims to determine whether the addition of SABR to systemic therapy will prolong survival compared with systemic therapy alone (axitinib ± immune checkpoint inhibitors (ICIs), lenvatinib ± ICIs, cabozantinib ± ICIs, sunitinib and pazopanib).

The phase III EA8211-SOAR trial (NCT05863351) similarly aims to determine whether SABR with systemic therapy is superior to systemic therapy alone, utilising a non-inferiority design comparing sequential SABR with all metastatic sites, with systemic therapy deferred until progression, against upfront standard-of-care systemic therapy; non-inferiority of SABR as first-line management of metastatic disease would represent robust level I evidence to support a systemic therapy-sparing strategy in oligometastatic RCC. Complementing this, the GETUG-StORM-01 trial (NCT04299646) and the Yale SBRT/ICI oligoprogression trial (NCT04974671) are addressing the related but distinct scenario of oligoprogression in established systemic therapy, where focal radiation to progressing lesions may allow continuation of an otherwise effective regimen and defer the need for a treatment switch.

RCC is characterised by a well-defined genetic background, with loss or mutation of the VHL gene occurring in over 70% of cases, and several oncogenic pathways—including VHL–HIF–VEGF angiogenesis signalling, PI3K/AKT/mTOR signalling, and epithelial-to-mesenchymal transition-related pathways—playing critical roles in tumour growth, invasiveness and metastasis. Emerging preclinical evidence has identified novel candidate biomarkers with potential relevance to both disease stratification and therapeutic targeting. Paraoxonase-2 (PON2), an intracellular membrane-bound enzyme whose upregulation has been reported across a range of malignancies, has recently been implicated in ccRCC biology; shRNA-mediated silencing of PON2 in ccRCC cell lines suppressed proliferation and migration while enhancing sensitivity to chemotherapeutic agents, suggesting its potential role as both a tumour survival mechanism and a targetable vulnerability [48]. Similarly, metabolic biomarkers identified through integrative omics approaches—including HIF-responsive markers such as carbonic anhydrase IX (CAIX)—are emerging as candidates to inform patient stratification and the selection of metabolic targeting strategies [49]. In the context of oligometastatic disease specifically, identifying biomarkers capable of distinguishing patients with indolent limited metastatic burden from those harbouring subclinical systemic dissemination may ultimately guide which patients are most likely to benefit from metastasis-directed therapy alone versus early systemic intervention.

## 10. Limitations

All included studies comprised patient cohorts that were predominantly male. While this reflects the higher global incidence of RCC in men, it limits the volume of sex-specific data available to inform management strategies that are equally applicable to women.

Another key limitation is that nearly all studies restricted inclusion to patients with an ECOG performance status of 0–1, representing a population with good baseline function. One of the principal advantages of locally directed therapies is their minimally invasive nature, making them particularly attractive for patients who are poor surgical candidates or unsuitable for metastasectomy. However, because frail or comorbid patients were largely excluded from the available literature, the efficacy and tolerability of these approaches in this clinically relevant population remain uncertain. Although SABR and cryoablation were generally well tolerated with low rates of adverse events, it is unclear whether these favourable outcomes can be extrapolated to patients with poorer performance status, in whom locally directed therapies may have the greatest real-world utility.

Clear-cell RCC, the most common histopathological subtype, predominated across the included studies. While this is representative of the general RCC population, evidence regarding the relative responsiveness of ccRCC to local therapies—particularly SABR—was inconsistent. Franzese et al. (2021) reported superior local control and survival for ccRCC compared with non-clear-cell subtypes in a large cohort treated with SABR for extracranial metastases [18], although the exact histological breakdown was not specified. Similarly, Zhang et al. (2019) observed improved survival among patients with ccRCC treated with SABR to all gross metastatic sites [37].

In contrast, Onal et al. found that ccRCC was associated with poorer or marginally worse OS in patients treated with SABR alone or in combination with TKIs, despite ccRCC comprising the majority of cases in both cohorts [31,32]. Smaller studies further highlight this variability: Ma et al. (*N* = 35) reported no progression among non-ccRCC lesions treated with SABR, albeit in a limited sample [26], whereas Stenman et al. (*N* = 117) found no significant association between histology, age, or tumour grade and OS, with brain metastases emerging as the only consistent adverse prognostic factor [34]. These findings should be interpreted with caution, as all of these studies are limited by small sample sizes and under-representation of non-clear-cell subtypes, limiting meaningful comparative analyses. Future studies should aim to stratify patients by histological subtype to enable more robust and reliable outcome analyses.

Finally, definitions of oligometastatic disease varied across studies, ranging from <3 to <6 metastatic lesions, with further discrepancies in definitions of outcomes including LC, PFS and FFLP across papers making outcomes difficult to compare and representing a limitation in the current state of the literature.

## 11. Conclusions

Oligometastatic RCC represents a distinct clinical state in which selected patients may derive meaningful benefit from locally directed therapies. The current evidence base most strongly supports the use of SABR, which consistently achieves high local control with acceptable toxicity across a range of metastatic settings, albeit largely limited to retrospective single-arm studies, and, therefore, represents a promising local treatment modality but cannot be definitively established as superior to systemic therapy, surveillance or other local therapies. Alternative local modalities, including radiofrequency ablation and cryoablation, appear safe and effective in carefully selected patients, although supporting data remain limited. In contrast, systemic therapies demonstrate more variable outcomes and are frequently associated with greater treatment-related morbidity. Importantly, existing studies predominantly include patients with good performance status, limiting the applicability of findings to broader and more comorbid populations.

Future research in oligometastatic RCC should prioritise prospective randomised trials with harmonised endpoints encompassing systemic-therapy-free survival and patient-reported quality of life, alongside biomarker-driven patient selection strategies and dedicated evaluation of emerging agents such as HIF-2α inhibitors and novel ICI combinations in the context of local metastasis-directed therapy.

## Figures and Tables

**Table 1:** Summary of included studies over the last 10 years investigating oligometastatic RCC with associated outcomes.

Author (Year)	Country	Study Type	Population	Performance Status	Histopathology	Intervention	Key Outcomes Reported	Median Follow Up	Main Findings/Conclusions/Prognosis Factors	Limitations
Allaf et al. (2024) [12]	USA/Canada	Randomised controlled trial (phase III)	819 patients with ≥T2 or Tany N+ RCC; No prior systemic or local therapy; ≤3 metastases; **excluding** brain, bone, and liver metastases	ECOG 0–1	CcRCC: 82% (intervention group); 84% (control) Mixed with ccRCC: 0% (intervention); 1% (control) Chromophobe: 7% (intervention); 6% (control)	Perioperative nivolumab (neoadjuvant + adjuvant) with two dosing schedules Control: nephrectomy + standard surveillance	Recurrence-free survival (primary)	Median follow up: 30.4 months (intervention); 30.1 months (control)	Primary endpoint not met; No RFS benefit in ITT population; In post hoc analysis of patients receiving >75% of planned nivolumab, no significant RFS advantage; High discontinuation (<50% completed full course) limited effective systemic exposure	Poor treatment adherence; Under-powered; Post hoc analyses; No placebo control; Protocol tolerance likely confounded efficacy assessment
Buti et al. (2020) [13]	Italy	Retrospective study	48 mRCC patients; 57 extracranial lesions Oligometastatic (≤5 lesions) or oligoprogressive (1–3 lesions); non-brain, non-bone-only lesions	Not specified	CcRCC: 93.7%; papillary: 6.3%	SABR to all treated lesions	**Primary:** Lesion progression-free survival (PFS); Radiologic response; Local control; Toxicity; Change in lesion diameter; Systemic therapy discontinuation (“treatment holiday”)	26.4 months	72.4% lesion PFS at 40 months; Median lesion PFS not reached; LC > 87%; 37.5% achieved ≥3.7-month systemic therapy holiday; Grade 1–2 AEs only, and no severe AEs; No difference in PFS if systemic therapy was continued vs. paused	Retrospective design; No control group; Progression defined radiologically; Heterogeneity in systemic therapy timing and continuation: continued or paused
Chow et al. (2018) [14]	USA	Retrospective + prospective cohort	145 patients with mRCC treated with HD IL-2 (retrospective *n* = 30; prospective *n* = 115); brain metastases excluded	ECOG 0–1	145 ccRCC; 1 papillary	High-dose IL-2 (two 5-day inpatient treatments per cycle; cycles repeated 12-weekly to response or intolerance) No control group;	ORR, CR rate, OS, and toxicity	Median follow-up: 39 months	ORR: 42.8%; CR: 20.7% (30/145); Median OS (entire cohort): 49.4 months; 58.1 months in favourable pathology cohort; 86.7% of CRs occurred in patients with 1–2 metastatic organ sites; Universal IL-2-related toxicities	Highly selected cohort; No control group; Potential selection bias (IL-2 only offered to fitter patients)
de Bruijn et al. (2017) [15]	The Netherlands	Retrospective study	40 patients with synchronous metastatic ccRCC and low-volume, incompletely resectable metastases following cytoreductive nephrectomy	ECOG < 2	Only ccRCC included	Observation post-nephrectomy; Selective delayed local therapy (EBRT, RFA, and metastasectomy) at progression	Time to progression (TTP); time to targeted therapy (TTT)	Unclear median follow-up	Median TTP: 6 months; Median TTT: 16 months; Median OS: 30 months; 25% received additional local therapy to further delay systemic treatment; Toxicity not specifically reported	Retrospective design; Small sample size; No control group; Heterogeneous metastatic sites and local treatments
Erdoğan et al. (2025) [16]	Turkey	Retrospective review	*N* = 42 patients with clear-cell mRCC and bone metastases	Not reported	All ccRCC	Surgical metastasectomy ± radiotherapy; Surgical management with or without **metastasectomy**; Systemic therapies including **targeted therapy (TKIs and mTOR inhibitors)**; **Radiotherapy** for bone metastases	OS, survival by metastatic burden, and skeletal site	Mean follow-up: 28.4 months	1-year OS: 73.7%; 2-year OS: 44.2%; 5-year OS: 13.7%; Solitary metastases and complete metastasectomy associated with longest survival; Axial skeleton involvement worse prognosis; Systemic therapy, radiotherapy, and antiresorptive therapy alone did not significantly improve OS; Toxicity not reported	Retrospective; Small sample size; Potential selection bias for surgical intervention; Limited prognostic performance of MSKCC and IMDC models in this cohort
Franzese et al. (2019) [17]	USA	Retrospective study	58 RCC patients, 73 metastatic lesions, and 25.8% prior metastasectomy; Oligometastatic (≤3 lesions); Primary tumour resected; Lung most common site	Not specified	ccRCC: 82.7%; papillary: 12%; chromophobe: 5.1%	SABR	LC; PFS	16.1 months	LC: 90.2% at 12 and 18 months; PFS: 46.2% at 12 months and 35% at 18 months; Metachronous disease and single metastasis predicted improved PFS; Prior systemic therapy improved LC in ccRCC	Retrospective design; Small sample size; Heterogeneous metastatic sites; Wide variation in follow-up duration (3.5–157 months); Non-standardized systemic therapy regimens
Franzese et al. (2021) [18]	Italy	Retrospective multicentre	207 RCC patients; 385 extracranial lesions; 245 SABR courses; Predominantly clear cell histology; Oligorecurrent (no ongoing systemic therapy) and oligoprogressive disease (isolated progression on systemic therapy or observation); extracranial only	ECOG 0–2	CcRCC: 84.1%; Papillary: 4.2%; Chromophobe: 2.6%; Spindle cell: 0.4%; Not defined: 2.8%	SABR; delivered with/without concurrent systemic therapy	Local control, PFS, progression risk, and toxicity	Median follow-up: 18.6 months	2 yr LC: 78.3%; Higher BED associated with improved LC and PFS; ccRCC showed greatest benefit Grade 1 acute toxicities only; no grade ≥3 events; No significant association between toxicity and treatment site or concurrent systemic therapy	Retrospective design; Heterogeneous systemic therapy; Inter-centre variation in SBRT technique/dose; Mix of oligoprogressive and oligorecurrent disease; No central imaging review; Potential classification bias
Franzese et al. (2022) [19]	Italy	Retrospective monocentre	129 oligometastatic RCC patients (≤5 metastases in ≤2 organs); 242 metastases; Brain most common site (34.7%), followed by lung (25.6%)	ECOG 0–2	CcRCC: 85.4%; Chromophobe: 1.22%; Papillary: 5.49%	SABR to metastases	Overall survival; Prognostic modelling	Median follow-up: **19.4 months**	Median OS: 43.6 months; 1, 2, and 3 yr OS: 82.6%, 64.7%, and 55.1%; Better OS with lung or nodal metastases; Worse OS with increasing age and brain metastases; Toxicity not specifically reported	Retrospective design, non-randomised with no comparator of systemic therapy alone; Single centre; Heterogeneous metastatic sites and systemic therapies
Franzese et al. (2023) [20]	Italy	Retrospective study	44 RCC patients; 57 SABR treatments; 74 oligoprogressive lesions (26 intracranial and 48 extracranial); All but one post-nephrectomy Oligoprogressive disease (≤5 lesions across ≤ 2 organs; cranial and extracranial)	ECOG 0–2	Not specified	SABR to oligoprogressive lesions (repeatable)	OS, PFS, and local failure	Median follow-up: **19 months**	1 yr OS: 79.2%; 2 yr OS: 57.3%; Median PFS: 9.8 months (1 yr: 43.2%; 2 yr: 25.8%); Improved PFS with longer disease-free interval and fewer treated lesions; Worse OS with brain metastases and multiple organs; Grade 1 and 2 AEs only	Heterogeneous concurrent and/or prior systemic therapy regimens; Small sample size; Mixed intracranial/extracranial sites
Gardner et al. (2017) [21]	USA	Retrospective cohort study	*N* = 40 metastatic RCC patients and 50 bone metastases; 25 oligometastatic patients (62.5%); 25 patients (62.5%) had **oligometastatic disease** (≤5 metastases)	Not reported	CcRCC: 70%; Mixed: 17.5%; Anaplastic: 2.5%; Chromophobe: 7.5%; Oncocytic: 2.5%	Cryoablation of bone metastases	Local tumour control per lesion, OS, and procedure-related complications	Median follow-up: 35 months	Overall LC: 82%; Oligometastatic patients: 96% vs. 53.3% in >5 metastases; Better control when ice-ball exceeded lesion diameter; 5 yr OS: 26%; Median survival in oligometastatic patients: 55.6 mo; 4 grade 3–4 AEs	Small sample size; Retrospective; Limited generalizability; No control group undergoing surgical metastasectomy
Gonnet et al. (2019) [22]	France	Retrospective cohort study	*N* = 53 metastatic RCC patients; ≤6 lung metastases; 28 had prior systemic therapy; A total of 100 lung metastases treated	Not reported	CcRCC: 90%; Papillary: 2%; Other: 8%	RFA to lung metastases; Repeat RFA allowed for recurrence	OS, DFS, pulmonary PFS, systemic treatment-free survival, local efficacy, and complications	Median follow-up: 61 months	5-year OS: 62%; Median DFS: 9.9 mo; 1 yr PPFS: 58.9%; 3 yr PPFS: 35.2%; Local efficacy: 91%; Median STFS: 28.3 months; T3/T4 primaries and ≥2 metastases associated with worse outcomes 3% grade 3 and 4 AEs	Retrospective; Selection bias toward RFA-eligible patients; Difficult to separate effect of RFA from systemic therapy
Hannan et al. (2022) [23]	USA	Prospective phase II single-arm clinical trial	23 systemic therapy-naïve mRCC patients; 33 initial extracranial lesions (57 treated with repeat SABR); Oligometastatic disease (≤3 extracranial metastases)	Not reported	Predominantly ccRCC: 82.6%; Papillary: 8.7%; Chromophobe: 8.7%	SABR to all oligometastatic sites	LC, PFS, time to systemic therapy, TTP, QoL, and toxicity	Median follow-up: 21.7 months	100% LC; 1 yr PFS: 82.6%; 1 yr freedom from systemic therapy: 91.3%; 1 yr TTP 87%; No QoL deterioration; SABR delivered prior to any systemic therapy delayed need for systemic treatment; Mostly grade 1 AEs; one grade 2; one grade 5 death; no grade 3–4 events	Small sample size; Single centre; Single-arm design; Short follow-up; Limited power for prognostic analyses
Hannan et al. (2022) [24]	USA	Prospective phase II single-arm trial	20 RCC patients with oligoprogressive disease on systemic therapy; 1–3 progressing sites comprising ≤30% of total metastatic burden	ECOG 0–2	Predominantly ccRCC: 90%; Papillary: 5%; NOS: 5%	SABR to progressing lesions (repeatable); Median systemic therapy duration combined with SABR compared with systemic therapy alone	LCl, duration of systemic therapy, QoL, and toxicity	10.4 months	100% LC; SABR extended ongoing systemic therapy by >6 months in 70% of patients (median extension: 11 months); Mostly grade 1–2; one grade 3 GI toxicity; no grade 4–5 events	Single-arm, non-randomised; Small sample size; Short follow-up period; Potential selection bias inherent to oligoprogression definitions
Lu et al. (2016) [25]	China	Retrospective cohort study	*N* = 67 patients with mRCC to bone treated with sutinib; *n* = 22 with oligometastatic bone metastases	ECOG 0–3	CcRCC: 88.1%; Non-ccRCC: 11.9%.	All patients underwent nephrectomy prior to sunitinib; Sunitinib (50 mg/day; 4 weeks on and 2 weeks off)	Median OS; MSKCC risk model	Unclear	Non-oligometastatic median OS: 12.7 months; Oligometastatic median OS: 30.1 months; Metastatic state, MSKCC score, ECOG and lymph node metastasis significantly associated with prognosis	Single centre; Small oligometastatic sample size; Heterogeneity of prior systemic treatments
Ma et al. (2022) [26]	China	Retrospective cohort	35 oligometastatic RCC patients (1–5 metastases)	ECOG 0–1	CcRCC: 82.9%; Papillary: 17.1; Other: 5.7%	SABR delivered as standard (small tumours) or partial (bulky or organ-adjacent lesions); Delivered before or with TKIs	PFS, OS, and toxicity	Median follow-up: **17 months**	Median PFS: 11.3 months; 1 yr PFS: 41.8%; 3 yr PFS: 27.9%; Median OS: 29.7 months; PFS markedly longer when all lesions irradiated (21.7 vs. 4.5 months); Non-ccRCC lesions showed no progression; Earlier RT associated with better outcomes; No grade ≥3 AEs	Retrospective; Small cohort size; Short follow up
Marvaso et al. (2021) [27]	Italy	Retrospective	61 mRCC patients; Intra- and extracranial disease; <5 metastases	Not reported	CcRCC: 75.4%; Others/unknown: 24.6%	SABR delivered post-nephrectomy or during systemic therapy; 18% received concurrent systemic therapy (TKIs or immunotherapy)	In-field PFS, out-of-field PFS, OS, and toxicity	Median follow-up: 2.3 years	In-field PFS: 70% at 1 year; 55% at 2 years; 1-year out-of-field PFS: 39%; 1-year OS: 78%; No > grade 1 toxicities	Retrospective design; heterogeneous patient population; variation in fractionation schedules; no non-radiotherapy control group
Meyer (2018) [28]	France	Retrospective cohort	188 mRCC patients; Oligoprogressive (*n* = 101), oligometastatic (*n* = 80), and residual disease post-systemic response (*n* = 7)	Not reported	CcRCC: 84.6%; Papillary: 3.2%; Other: 3.2%; Unknown: 9.0%	SABR to metastases	Local control, PFS, and OS	Median follow-up: 22 months	LC: 87.5% at 6 months, 82.9% at 12 months, and 77.6% at 24 months; Median PFS: 8.5 months overall (OP: 8.6; OM: 7.6); Median OS: 29.2 months (OP: 23.2; OM: 33.9) Mostly grade 1–2 toxicity (*n* = 54), five grade 3 AEs, and no grade 4–5 AEs	Retrospective; heterogeneous dose; fractionation schedules; heterogeneous patient population and indications; effect of concurrent systemic therapy not evaluated
Miller et al. (2016) [29]	USA	Retrospective cohort study	*N* = 100 patients with RCC spinal metastases treated with stereotactic radiosurgery	Not specified	80% ccRCC	Spine SRS (median: 16 Gy ×1 fraction)	Local failure at 12 months	Median follow-up ranged from 6 months (cohort D) to 18 months (cohort E)	12-month local failure: 4% with concurrent first-line TKI + SRS; 19–27% in other SRS cohorts; 57% in negative control group 46% received concurrent TKI at time of SRS; No grade ≥3 adverse events reported	Retrospective; Selection and measurement bias; Differences in baseline survival between cohorts; Heterogeneous systemic therapy exposure
Onal et al. (2022) [30]	Turkey	Retrospective cohort	54 oligometastatic RCC patients (≤5 metastases); Predominantly spinal lesions (57.4%); Single metastasis in 64.8%	Not reported	CcRCC: 77.8%; Papillary: 9.3%; Chromophobe: 9.3%; Unclassified: 3.6%	SABR to metastases	OS, PFS, and LC	Median follow-up: 22.4 months	Median OS: 43.1 months; 1 and 2 yr OS: 84.6% and 67.3%; Median PFS: 15.3 months; 1 yr LC: 94.9%; Spinal metastases and single-fraction SBRT associated with improved OS; Progression predominantly distant No grade ≥ 3 AEs	Retrospective; Small patient population; Selection bias; Heterogeneous population with varying fractionation schedules; No control group of oligometastatic RCC patients without bone metastases
Onal et al. (2022) [31]	Turkey	Retrospective cohort	70 oligometastatic RCC patients (≤5 metastases); Intracranial excluded; Single metastasis in 65.7%	ECOG 0–1	CcRCC: 65.7%; Chromophobe: 12.9%; Papillary: 7.1%; Unclassified: 4.3%	SABR to metastases	OS, PFS, and progression patterns	Median follow-up: 21.1 months	Median OS: 49.1 months; Median PFS: 18.3 months; 1 yr OS: 81.9%; 1 yr: PFS 64.9%; 50% progressed at median of 12.9 months; CcRCC associated with poorer OS 15.7% grade 1 AEs; no grade ≥ 3 AEs	Small patient population; Retrospective; Heterogeneous fractionation schedules; No non-SABR control group; Mostly bone and lung metastases
Onal et al. (2023) [32]	Turkey	Retrospective cohort study	*N* = 42 RCC patients with ≤5 metastases	ECOG 0–1	CcRCC: 73.8%; Non-ccRCC: 26.2%	SABR (≥5 Gy/fraction, BED ≥ 90 Gy)	OS, PFS, LC, and systemic therapy modification	Median follow-up: **62.3 months**	Median OS: 30.5 mo; 2-year OS: 58%; 2-year local control: 94.1%; PFS: 51.3%; 60% developed distant metastases; SABR delayed systemic therapy modification in most patients Mostly grade 1–2 AEs; one grade 3 AE CcRCC marginally worse OS compared with non-ccRCC;	Small sample size; Retrospective; Heterogeneous fractionation schedules; No control group treated with TKI alone
Siva et al. (2022) [33]	Australia	Single-arm, multi-institutional Phase I/II trial	*N* = 30 patients with ccRCC; 1–5 metastases;	ECOG 0–2	All ccRCC	SABR to all metastatic sites (20 Gy ×1 fraction or 10 ×3 Gy) followed by pembrolizumab (200 mg IV q3w ×8 cycles)	AEs (primary endpoint), ORR, PFS, and OS	Median follow-up: 28 months	ORR: 63%; 1-year PFS: 60%; 2-year PFS: 45%; 1-year OS: 90%; 2-year OS: 74%; Grade 3 AEs: 13%; Grade 1–2: 63%; no AEs: 23%	Single-arm design; Small sample size; No comparator group; Limited follow up
Stenman et al. (2018) [34]	Sweden	Retrospective cohort study	*N* = 117 metastatic RCC patients; 86% ccRCC	ECOG 0–1	CcRCC 86% in SABR group, 83% in SM group, and 97% in both modality group; Remainder papillary, chromophobe, and other/unknown	SABR (*n* = 57), surgical metastasectomy (*n* = 30), sequential SABR+ surgery (*n* = 30), and other local ablative therapies (RFA *n* = 3; IRE *n* = 1)	OS, impact of local therapy modality, and clinicopathologic survival factors	Median follow-up: 63 months	Median OS: 51 mo; No significant OS differences between SRT, surgery, or combined approaches; Brain metastases associated with worse survival; Other organ involvement, age, tumour grade, and histology did not impact OS	Retrospective study design; Selection bias in treatment allocation; Heterogeneity in number of metastases and treatment sequences; Lack of randomisation and control for systemic therapies
Tang et al. (2025) [35]	USA	Prospective phase 2 trial	121 oligometastatic ccRCC patients (1–5 metastases); 118 post-nephrectomies; 36 previously received systemic therapy	ECOG 0–2	Only ccRCC included	SABR to all lesions	PFS, OS, systemic therapy-free survival, and toxicity	Median follow-up: 36.3 months	Median PFS: 17.7 months; Median systemic therapy-free survival: 34.0 months; 1 yr OS: 96.7%; 3 yr: OS 86.5%; High LC; SABR used to delay initiation or re-initiation of systemic therapy; Grade 3–4 AEs in 7%; Grade ≥ 2 toxicities in 21%	Single institution; Non-randomized; Potential selection bias
Udovicich et al. (2025) [36]	Australia	Retrospective	34 RCC patients; All subtypes included: 91% ccRCC; Synchronous, metachronous, oligoprogressive and oligopersistent disease	Not reported	CcRCC: 91%; Non-ccRCC: 9%	SABR: *n* = 28; Conventional fractionation; RT: *n* = 18; Surgical resection: *n* = 4 (7%)	PFS, FFLP, and patterns of failure	Median follow-up: 4.1 years	FFLP: 94% at 1 yr and 85% at 3–5 yrs; PFS: 47% at 1 yr, 26% at 3 yrs, and 8% at 5 yrs; Majority of first failures distant Incidence of first failure (distant alone): 44% at 1 year and 62% at 3 years; Incidence of first failure (death alone or synchronous local + distant progression): 3% at 1 and 3 years;	Retrospective; Small sample size; Heterogeneous population including non-ccRCC subtypes; Imaging heterogeneity; Use of two different PSMA tracers
Zhang et al. (2019) [37]	USA	Retrospective study	47 oligometastatic RCC patients	Not reported	41 ccRCC; 6 non-ccRCC	SABR to all gross metastatic sites; Most received systemic therapy at progression	OS, FST, LC, and toxicity	Median follow-up: 30 months	Median FST: 15.2 months; 1 yr OS: 93.1%; 2 yr OS 84.8%; Patients with single metastasis had superior FST; No grade ≥ 3 toxicity; Better survival associated with favourable risk, ccRCC, and absence of metastatic disease at diagnosis	Retrospective; Single institution; Relatively short follow-up; Selection bias in patient eligibility for SABR

Abbreviations: AE = adverse event; BED = biologically effective dose; ccRCC = clear-cell renal-cell carcinoma; DFS = disease-free survival; FFLP = freedom from local progression; FST = freedom from systemic therapy; HD IL-2 = high-dose interleukin-2; LC = local control; ORR = objective response rate; OS = overall survival; PFS = progression-free survival; PSMA = prostate-specific membrane antigen; RFA = radiofrequency ablation; RFS = recurrence-free survival; SABR = stereotactic ablative body radiotherapy; SM = surgical metastasectomy; SRS = stereotactic radiosurgery; TKI = tyrosine kinase inhibitor.

**Table 2:** Summary of current guidelines regarding definitions and treatment of oligometastatic RCC.

Guideline	Year	Oligometastatic Definition	Local/MDT	Systemic Therapy	Oligoprogression	Patient Selection Criteria
EAU	2025	Not explicitly defined; defers to ESTRO–ASTRO consensus (1–5 lesions)	Metastasectomy or SABR recommended for bone or brain metastases; Observation recommended for unresectable oligometastases prior to initiation of systemic therapy	Standard ICI ± TKI per IMDC risk group; no oligomRCC-specific systemic recommendation provided	Not explicitly addressed	Resectability of lesions; bone or brain metastatic site as primary driver of local treatment selection
NCCN	2026	Not formally defined; clinical context used	Metastasectomy, SABR, or ablative techniques for oligometastatic or oligoprogressive disease	Standard systemic therapy per IMDC risk group for patients not suitable for local treatment; Adjuvant pembrolizumab considered post-resection in high-risk ccRCC	Local ablative treatment to progressing lesions to extend benefit of ongoing systemic therapy	Good performance status; limited metastatic burden; lesion amenability to local treatment
ASCO	2023	Implicit; favourable/intermediate IMDC risk with limited metachronous disease	Complete metastasectomy or stereotactic RT to all sites recommended to achieve disease control and delay systemic therapy; Adjuvant pembrolizumab may be considered following resection in selected patients with ccRCC histology	Standard ICI-based combination therapy recommended for patients unsuitable for upfront local therapy; agent selection per IMDC risk	Local ablative treatment to progressing lesions may prolong benefit of ongoing systemic therapy; supports treatment switch deferral	Favourable or intermediate IMDC risk; good performance status; limited, metachronous disease; no high-risk metastatic sites
AUA	2021	Not defined; no dedicated oligometastatic RCC guidelines	Surgical or ablative approaches may be considered following appropriate disease staging in selected patients	Standard systemic therapy per guideline risk stratification; no oligometastatic RCC-specific systemic recommendations provided	Not addressed	Appropriate disease staging required prior to any local intervention; no specific patient selection criteria stated
KCRNC	2021	Not formally defined; references limited metastatic burden in clinical context	Metastasectomy, radiotherapy, or ablative techniques (including SABR) endorsed for oligometastatic disease	Standard ICI ± TKI per IMDC risk group; systemic therapy recommended when local treatment is not feasible	Local ablative treatment endorsed for oligoprogressive disease to extend the benefit of ongoing systemic therapy	Limited metastatic burden; lesion amenability to local treatment; performance status not explicitly stated

Abbreviations: ASCO = American Society of Clinical Oncology; AUA = American Urological Association; ccRCC = clear-cell renal-cell carcinoma; EAU = European Association of Urology; ESTRO–ASTRO = European Society for Radiotherapy and Oncology-American Society for Radiation Oncology; ICI = immune checkpoint inhibitor; IMDC = International Metastatic Renal-Cell Carcinoma Database Consortium; KCRNC = Kidney Cancer Research Network of Canada; MDT = metastasis-directed therapy; NCCN = National Comprehensive Cancer Network; RT = radiotherapy; SABR = stereotactic ablative body radiotherapy; TKI = tyrosine kinase inhibitor.

## Data Availability

This narrative review did not generate or analyse new datasets. All data supporting the findings of this study are contained within the cited published literature.

## Abbreviations

The following abbreviations are used in this manuscript:

RCC	Renal-cell carcinoma
SABR	Stereotactic ablative body radiotherapy
ccRCC	Clear-cell renal-cell carcinoma
mRCC	Metastatic RCC
RFA	Radiofrequency ablation
PD-1	Programmed cell death protein 1
CTLA-4	Cytotoxic T lymphocyte-associated protein 4
TKIs	Tyrosine kinase inhibitors
ECOG	Eastern Cooperative Oncology Group
BED	Biologically effective dose
PFS	Progression-free survival
OS	Overall survival
PSMA	Prostate-specific membrane antigen
PET	Positron emission tomography
CT	Computed tomography
FFLP	Freedom from local progression
CTCAE	Common Terminology Criteria for Adverse Events
AE	Adverse event
FST	Freedom from systemic therapy
SM	Surgical metastasectomy
IL-2	Interleukin-2
SRS	Stereotactic radiosurgery
ASMase	Acid sphingomyelinase
VHL	von Hippel–Lindau
MDT	Metastasis-directed therapy
